# Supplementary material for: Intra Articular Injection of Autologous Microfat and Platelets-Rich Plasma in the Treatment of Wrist Osteoarthritis: A Pilot Study
Source: J Clin Med. 2022 Sep 29;11(19):5786. doi: 10.3390/jcm11195786 (PMC9572253; doi:10.3390/jcm11195786)
Supplement: Supplementary file 1 [file jcm-11-05786-s001.zip › jcm-1839618-supplementary.pdf]

Supplementary Materials

Supplementary Table S1. Patient-related Outcomes over the 12 months post treatment

	Mean ± SD or n (%)	Mean Change ± SD	Median Change (min – max)	Adjusted p-value
PAIN VAS				
Baseline	58.3 ± 13.2			
1 months	35.8 ± 17.3	-22.5 ± 19	-20 (-57 – 0)	0.008
3 months	32.7 ± 13.4	-25.6 ± 14.7	-20 (-49 – 0)	0.003
6 months	25.4 ± 11.4	-32.8 ± 12.9	-30 (-50 – -15)	0.002
12 months	25.8 ± 15.2	-32.4 ± 17.8	-32.5 (-64 – 0)	0.003
Pain VAS MCID 14 points				
1 months	8 (66.7)			0.248
3 months	10 (83.3)			0.021
6 months	12 (100)			<0.001
12 months	11 (91.7)			0.004
Pain VAS > 30 points				
1 months	4 (33.3)			0.248
3 months	4 (33.3)			0.248
6 months	8 (66.7)			0.248
12 months	7 (58.3)			0.564
DASH				
Baseline	44.5 ± 15.3			
3 months	30.3 ± 15.8	-14.2 ± 12	-11.3 (-39.2 – 2.4)	0.002
6 months	27.3 ± 14.4	-17.2 ± 12.6	-17.9 (-39.2 – 5.7)	0.002
12 months	24.1 ± 16.6	-20.4 ± 11.8	-23 (-35 – -0.1)	0.002
DASH responder (10.83 points)				
3 months	7 (58.3)			0.564
6 months	10 (83.3)			0.021
12 months	9 (75)			0.083
PRWE				
Baseline	62 ± 19.1			
3 months	32.8 ± 17	-29.2 ± 17.9	-25 (-67.5 – -8)	0.001

6 months	29.5 ± 16.6	-32.4 ± 21.2	-29.8 (-67.5 – 1.5)	0.002
12 months	26.3 ± 20.8	-35.7 ± 19.5	-32.5 (-67 – -7.5)	0.001
PRWE responder (11.5 points)				
3 months	11 (91.7)			0.012
6 months	10 (83.3)			0.021
12 months	11 (91.7)			0.012

Median of absolute change from baseline. Adjusted P value: Wilcoxon signed rank test with Stepdown Bonferroni correction. Test of Binomial proportion (p=0.5).

Supplementary Table S2: Clinical measures over the 12 months post treatment.

		Mean ± SD	Mean Change ± SD	Median Change (min – max)	Adjusted p-value
Wrist strength (kg)	Baseline	25.9 ± 9.6			
injured wrist	3 months	29.8 ± 10.7	3.9 ± 2.9	4.2 (-1 – 8.7)	0.005
	6 months	33.4 ± 12.3	7.5 ± 6.7	7.7 (-2.3 – 20)	0.005
	12 months	34.2 ± 12.4	8.3 ± 6.9	7.3 (0.7 – 24)	0.002
	Baseline	38.8 ± 12.8			
normal wrist	3 months	41.4 ± 12.5	2.6 ± 4.4	1.3 (-3.3 – 13.3)	0.054
	6 months	42.7 ± 13.5	3.9 ± 5.7	4.7 (-8.7 – 12.7)	0.054
	12 months	44.2 ± 12.7	5.4 ± 4.9	2.7 (-1.3 – 15.3)	0.032
Extension (°)	Baseline	40.3 ± 12.4			
injured wrist	3 months	35.8 ± 9.6	-4.4 ± 8.6	-3 (-24 – 6)	0.404
	6 months	40 ± 11.6	-0.3 ± 10.1	2 (-26 – 14)	0.945
	12 months	39.8 ± 9.3	-0.4 ± 6.2	0 (-14 – 10)	0.945
	Baseline	64.3 ± 16.5			
normal wrist	3 months	61.8 ± 14.7	-2.4 ± 15.8	-0.5 (-30 – 22)	0.756
	6 months	60.3 ± 13	-3.9 ± 13.4	-1.5 (-40 – 10)	0.756
	12 months	60.2 ± 17.9	-4.1 ± 20.3	-1 (-62 – 20)	0.756

Flexion (°)	Baseline	33.8 ± 10.5			
injured wrist	3 months	36.8 ± 14.7	2.9 ± 11.9	3 (-15 – 32)	0.973
	6 months	37.5 ± 9.4	3.7 ± 10.9	2 (-18 – 20)	0.750
	12 months	33.4 ± 13.8	-0.4 ± 9.8	-1 (-19 - 14)	0.973
normal wrist	Baseline	55.8 ± 9.9			
	3 months	59.3 ± 15.5	3.6 ± 13.9	1 (-17 – 40)	0.875
	6 months	55.3 ± 8.2	-0.4 ± 6.9	0 (-9 – 14)	0.875
	12 months	52.5 ± 12.3	-3.3 ± 13.3	-2 (-37 – 18)	0.875
Ulnar inclination (°)	Baseline	15.9 ± 7.1			
injured wrist	3 months	20 ± 4.7	4.1 ± 7.9	3.5 (-10 – 18)	0.106
	6 months	23.8 ± 4.7	7.9 ± 8.4	8 (-10 – 22)	0.031
	12 months	22.5 ± 6.4	6.6 ± 7.2	7 (-6 – 18)	0.031
normal wrist	Baseline	31.9 ± 11.1			
	3 months	30.2 ± 6.5	-1.8 ± 9.6	-2 (-20 – 16)	0.999
	6 months	31.8 ± 10.2	-0.1 ± 12.2	-4 (-18 – 20)	0.999
	12 months	28.7 ± 9.4	-3.3 ± 10.3	-3 (-20 – 14)	0.999
Radial inclination (°)	Baseline	12.2 ± 3.6			
injured wrist	3 months	16.7 ± 5.6	4.5 ± 6.1	4 (-8 – 17)	0.077
	6 months	14.7 ± 5.2	2.5 ± 4.6	1.5 (-6 – 10)	0.195
	12 months	13.9 ± 6.9	1.8 ± 6.4	0.5 (-6 – 13)	0.561
normal wrist	Baseline	23.4 ± 9			
	3 months	23.5 ± 8.1	0.1 ± 9.7	0 (-12 – 20)	0.896
	6 months	24 ± 7.9	0.6 ± 11.7	1 (-20 – 23)	0.896
	12 months	24 ± 8.7	0.6 ± 12	0 (-18 – 22)	0.896

Median of absolute change from baseline. Adjusted P value: Wilcoxon signed rank test with Stepdown Bonferroni correction.

Supplementary Table S3. Patient-related Outcomes over the 12 months post treatment by localization

	**RC patient n=8**			Test of Change from baseline	**RC+MC patients n=4**			Comparison Between groups
	Mean ± SD or n (%)	Mean Change ± SD	Median Change (min – max)	Adjusted p-value	Mean ± SD or n (%)	Mean Change ± SD	Median Change (min – max)	Adjusted p-value
PAIN VAS								
Baseline	57.5 ± 13.9				59.8 ± 13.7			
1 months	32.1 ± 18.4	-25.4 ± 14.8	-22 (-49 - 0)	0.047	43 ± 14	-19.8 ± 28.1	-29.5 (-40 - 20)	0.343
3 months	32.8 ± 16.5	-24.8 ± 15.8	-24.5 (-49 - 0)	0.047	32.5 ± 5	-16.8 ± 27.2	-5 (-57 - 0)	1.000
6 months	21.9 ± 12.5	-35.6 ± 11	-35 (-50 - -20)	0.039	32.5 ± 2.9	-27.3 ± 14.5	-20 (-49 - -20)	0.303
12 months	23.8 ± 16	-33.8 ± 13.4	-35 (-51 - -19)	0.039	30 ± 14.7	-27.3 ± 16.1	-22.5 (-49 - -15)	0.797
Pain VAS MCID 14 points								
1 months	7 (87.5)				1 (25)			0.200
3 months	6 (75)				4 (100)			0.667
6 months	8 (100)				4 (100)			NA
12 months	8 (100)				3 (75)			0.667
Pain VAS > 30 points								
1 months	3 (37.5)				1 (25)			1.000
3 months	3 (37.5)				1 (25)			1.000
6 months	6 (75)				2 (50)			1.000
12 months	5 (62.5)				2 (50)			

	RC patient n=8			Test of Change from baseline	RC+MC patients n=4			Comparison Between groups
	Mean ± SD or n (%)	Mean Change ± SD	Median Change (min – max)	Adjusted p-value	Mean ± SD or n (%)	Mean Change ± SD	Median Change (min – max)	Adjusted p-value
DASH								
Baseline	44.4 ± 16.6				44.6 ± 14.6			
3 months	29.9 ± 19.5	-14.5 ± 12.9	-9.6 (-39.2 - -3.3)	0.023	31 ± 5.1	-13.6 ± 11.8	-17.1 (-22.5 - 2.4)	0.932
6 months	24 ± 15.7	-20.4 ± 12.4	-20.4 (-39.2 - 0.8)	0.023	34 ± 9.9	-10.7 ± 11.7	-13.8 (-20.8 - 5.7)	0.234
12 months	22 ± 19.1	-22.4 ± 12.4	-26.7 (-35 - -3.3)	0.023	28.3 ± 11.2	-16.3 ± 11.1	-20.4 (-24.2 - -0.1)	0.350
DASH responder (10.83 points)								
3 months	4 (50)				3 (75)			1.000
6 months	7 (87.5)				3 (75)			1.000
12 months	6 (75)				3 (75)			1.000
PRWE								
Baseline	60.9 ± 19.5				64.1 ± 20.9			
3 months	32.6 ± 20.4	-28.3 ± 18.2	-21.5 (-67.5 - -11.5)	0.0234	33.1 ± 9.4	-31 ± 20	-30 (-56 - -8)	0.799
6 months	25 ± 16.9	-35.9 ± 22.4	-38 (-67.5 - 1.5)	0.0234	38.6 ± 13.6	-25.5 ± 19.6	-26.5 (-48.5 - -0.5)	0.552
12 months	22.8 ± 22.4	-38.1 ± 18.6	-37.5 (-67 - -12.5)	0.0234	33.3 ± 17.8	-30.9 ± 23.2	-26.8 (-62.5 - -7.5)	0.445
PRWE responder (11.5 points)								
3 months	8 (100)				3 (75)			1.000
6 months	7 (87.5)				3 (75)			1.000
12 months	8 (100)				3 (75)			1.000

Median of absolute change from baseline. Adjusted P value: Wilcoxon signed rank test with Stepdown Bonferroni correction. Test of Binomial proportion (p=0.5).

Supplementary Table S4: Clinical measures over the 12 months post treatment by localization

		**RC patient n=8**			Test of Change from baseline	**RC+MC patients n=4**			Comparison Between groups
		Mean ± SD	Mean Change ± SD	Median Change (min – max)	Adjusted p-value	Mean ± SD or n (%)	Mean Change ± SD	Median Change (min – max)	Adjusted p-value
Wrist strength (kg)	Baseline	27.9 ± 10.3				21.9 ± 7.7			
injured wrist	3 months	32.2 ± 12.1	4.3 ± 3.1	4.3 (-1 - 8.7)	0.023	25.2 ± 5.4	3.3 ± 2.8	4.2 (-0.7 - 5.3)	0.799
	6 months	37.8 ± 11.5	9.9 ± 6	9.7 (1 - 20)	0.023	24.5 ± 9.4	2.6 ± 5.6	2 (-2.3 - 8.7)	0.075
	12 months	38.5 ± 11.6	10.5 ± 7.3	8 (2.3 - 24)	0.023	25.8 ± 10.4	3.8 ± 3.4	3 (0.7 - 8.7)	0.148
	Baseline	40.3 ± 14.2				35.8 ± 10.6			
normal wrist	3 months	44.1 ± 13.8	3.8 ± 4.7	2.3 (-0.7 - 13.3)	0.094	36 ± 8.3	0.2 ± 2.7	0.3 (-3.3 - 3.3)	0.234
	6 months	46 ± 14.6	5.8 ± 3.9	5.7 (-0.7 - 12.7)	0.047	36 ± 9.1	0.2 ± 7.6	0.7 (-8.7 - 8)	0.268
	12 months	48.2 ± 12.4	5 ± 5.8	3.3 (-1.3 - 15.3)	0.094	37.3 ± 11.4	1.5 ± 1.8	1.7 (-0.7 - 3.3)	0.298

Median of absolute change from baseline. Adjusted P value: Wilcoxon signed rank test with Stepdown Bonferroni correction
